# Nitric Oxide Donor Metallodrug: Single-Inhaler Proposal for Rescue in Acute Allergic Asthma Crises

**DOI:** 10.3390/biology14030244

**Published:** 2025-02-27

**Authors:** Paula Priscila Correia Costa, Stefanie Bressan Waller, Hálef Herbet Ramos, Belarmino Eugênio Lopes Neto, Wesley Lyeverton Correia Ribeiro

**Affiliations:** 1 Department of Veterinary Clinics, Faculty of Veterinary, Federal University of Pelotas (UFPEL), Pelotas 96010-610, RS, Brazil; waller.stefanie@yahoo.com.br; 2 Department of Physiology and Pharmacology, Faculty of Medicine, Federal University of Ceará (UFC), Fortaleza 60355-636, CE, Brazil; wesleylyeverton@yahoo.com.br; 3 Department of Social Medicine, Faculty of Medicine, Federal University of Pelotas (UFPEL), Pelotas 96010-610, RS, Brazil; haleframos@yahoo.com.br; 4 Western College of Veterinary Medicine, University of Saskatchewan, Saskatoon, SK S7N 5A2, Canada; belarminovet@gmail.com

**Keywords:** nitrosyl complex, airway inflammation, asthma crisis, acute response, bronchial hyperreactivity, rescue therapy, immune modulation, smooth muscle relaxation, rapid relief, murine model

## Abstract

Allergic asthma is characterized by chronic airway inflammation and recurrent bronchial hyperreactivity, highlighting the need for rapid therapeutic interventions during acute crises. This study aimed to assess the potential of a single-dose administration of the ruthenium nitrosyl complex *cis*-[Ru(bpy)_2_(2-MIM)(NO)](PG_6_)_3_ (named as FOR811A) as a fast-acting treatment in a murine model of allergic asthma. Female Swiss mice were sensitized with ovalbumin for the induction of asthma and subjected to inhalation challenges. The experimental groups included controls and ovalbumin-sensitized mice receiving FOR811A (0.75 mg/kg) or saline (NaCl 0.9%), both by gavage. Lung tissues were collected for analyses of oxidative damage (nitrite/nitrate and GSH), inflammatory markers (myeloperoxidase, IL-1β, and IL-4), and histological assessment. The results showed that, while FOR811A did not significantly reduce oxidative damage or overall inflammation, it effectively decreased IL-4 levels, indicating a modulation of the Th2 immune response without affecting IL-1β levels (Th1 response). These findings suggest that a single-dose administration of FOR811A may provide a rapid therapeutic effect in allergic asthma crises by promoting smooth muscle relaxation and modulating immune responses. Further research is warranted to explore its clinical utility as a fast-acting rescue medication for acute asthma management.

## Simple Summary

Allergic asthma is a widespread condition characterized by airway inflammation, causing breathing difficulties, wheezing, cough, and chest tightness. This study explores the potential of a new treatment using a compound called FOR811A, designed to provide quick relief during asthma attacks. Researchers tested this compound on mice that had been sensitized to allergens. The aim was to see if a single dose could help reduce inflammation and improve breathing. While the treatment did not significantly decrease overall inflammation or damage, it was effective in lowering specific immune markers associated with allergic reactions. This suggests that FOR811A could help relax the muscles in the airways during an asthma crisis, offering a promising option for rapid relief. These findings are important, as they offer further insights into managing allergic asthma, a prevalent condition that can lead to serious complications if not effectively controlled. Developing fast-acting treatments like FOR811A could greatly improve the quality of life for those living with asthma, making it easier to control their symptoms during critical moments.

## 1. Introduction

Asthma is a worldwide public health issue, affecting the sufferer’s quality of life [1], characterized by chronic inflammation of the airways and common respiratory symptoms such as wheezing, shortness of breath, chest tightness, and coughing [2]. This respiratory disease is heterogeneous and complex, featuring various phenotypes (observable characteristics of an individual) and endotypes (underlying molecular or pathophysiological mechanisms) [3]. Its diagnosis involves identifying the characteristic pattern of respiratory symptoms and distinguishing asthma from other respiratory conditions, such as chronic upper airway cough syndrome, cystic fibrosis, bronchiectasis, pulmonary embolism, and inhaled foreign bodies, among others [2].

According to the Global Initiative for Asthma, asthma control has the following two domains: symptom control and the future risk of adverse outcomes [2]. Pharmacological management requires a personalized approach, including medications, patient education, and periodic reviews of inhalation technique [3]. In general, asthma treatment uses inhaled corticosteroids (ICSs), with or without a long-acting beta-2 agonist (LABA, e.g., formoterol), while short-acting bronchodilators (SABAs, e.g., salbutamol [albuterol], terbutaline) and ipratropium are indicated as rescue medications during the stages of treatment [2,3].

However, rescue inhalers should not be used for regular maintenance or in the absence of symptoms, except before exercise [2]. Some medications, such as ipratropium, can lead to adverse effects, including nausea and tremors [2], as well as an increased risk of arrhythmias [4]. In addition to tachycardia, tremors, and headache, the use of SABAs can cause more severe effects, such as sudden constriction of the bronchial airways or paradoxical bronchospasm, hypokalemia, and myocardial infarction [5]. Furthermore, salbutamol and formoterol have recently been associated with the risk of causing supraventricular tachycardia [6]. Furthermore, treatment responses vary among patients, necessitating the search for new drugs that provide faster and more effective respiratory relief, thereby improving quality of life.

As an alternative, it is known that metals play essential roles in nutrients and medicines, offering chemical functionalities that are absent from organic compounds. Used in therapies as metallodrugs, these substances are often prodrugs activated by ligand substitution or redox reactions and have multiple targets, aspects that must be considered when establishing structure–activity relationships [7].

Recently, our group showed that the metallodrug nitrosyl-ruthenium *cis*-[Ru(bpy)_2_(2-MIM)(NO)](PG_6_)_3_, named FOR811A, was promising in a murine model of allergic asthma by relaxing the bronchial smooth muscles and improving respiratory mechanics [8]. Testing candidate compounds for reliever medications is crucial to identify effective treatments that can provide rapid relief from asthma symptoms while minimizing adverse effects.

Given the potential of metallodrugs, this study aimed to evaluate the effects of FOR811A as a rapid-acting treatment in a murine model of allergic asthma as a proposal for a single inhaler for rescue in asthma crises.

## 2. Materials and Methods

### 2.1. Synthesis of Ruthenium Complex and Preparation for In Vivo Use

**Figure 1:**
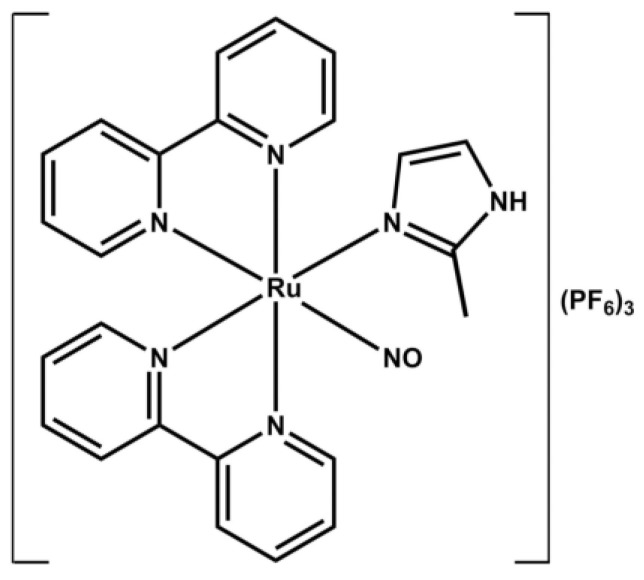
Chemical structure of FOR811A, a metallodrug based on the ruthenium complex *cis*-[Ru(bpy)_2_(2-MIM)(NO)](PF_6_)_3_ [9].

The ruthenium compound *cis*-[Ru(bpy)_2_(2-MIM)(NO)](PF_6_)_3_ complexed with the NO molecule (Figure 1) was synthesized at the Bioinorganic Laboratory (LABIO) of the Department of Organic and Inorganic Chemistry at the Federal University of Ceará (UFC, Fortaleza, CE, Brazil), according to the method described by Gouveia-Júnior [9]. This metallodrug was designated as FOR811A and was previously tested by our team, demonstrating a proven anti-asthmatic effect [8].

For in vivo use as a rescue drug, 40 mg of the metallodrug FOR811A was dissolved in 8 mL of a sterile saline solution (0.9% NaCl). This preparation was made immediately prior to administering the gavage to the animals.

### 2.2. Experimental Animals

Female Swiss mice (*Mus musculus*, *n =* 40), aged from 14 to 16 weeks and weighing between 25 and 30 g, were used in the study. The animals were sourced from the Central Animal Facility of the Federal University of Ceará (BIOCEN-UFC, Fortaleza, CE, Brazil) and housed in the animal facility of the Institute of Biomedical Sciences of the State University of Ceará (UECE, Fortaleza, CE, Brazil). They were kept in groups of four per cage, under a 12 h light/dark cycle, at a temperature of 22 °C. Food and water were provided *ad libitum*. All experimental protocols involving the use of animals were previously approved by the Ethics Committee on Animal Use (CEUA) of the State University of Ceará (UECE), under protocol 2068307/2018.

### 2.3. Experimental Design and Asthma Induction Protocol

The study consisted of four experimental groups (*n* = 10 each), designated as follows: a control group treated with saline (Ctl+Sal), a control group treated with the metallodrug FOR811A (Ctl+FOR), an asthmatic group treated with saline (Ast+Sal), and an asthmatic group treated with FOR811A (Ast+FOR).

On days 0, 7, and 14, the animals in the control group were sensitized by an intraperitoneal injection of saline solution (0.9% NaCl, groups Ctl+Sal and Ctl+FOR), while the animals in the asthmatic groups (Ast+Sal and Ast+FOR) were induced to asthma through an intraperitoneal injection of ovalbumin (OVA, 100 μg), dissolved in aluminum hydroxide ([Al(OH)_3_], 5 mg).

On days 26, 27, and 28, the animals were challenged by being placed in an acrylic chamber (30 cm × 15 cm × 20 cm) connected to an ultrasonic nebulizer (US–1000, ICEL, São Paulo, Brazil) for the inhalation of 0.9% NaCl for 20 min (groups Ctl+Sal and Ctl+FOR) or the inhalation of 1% OVA (10 mg/mL) for 20 min (groups Ast+Sal and Ast+FOR).

On day 29, the animals were treated orally (0.2 mL by gavage) with a single dose of 0.9% NaCl (groups Ctl+Sal and Ast+Sal) or FOR811A at 0.75 mg/kg (groups Ctl+FOR and Ast+FOR).

Finally, on day 30, euthanasia was performed through an overdose of anesthetics, using a combination of 10% ketamine hydrochloride (300 mg/kg, Cetamin, Syntec, São Paulo, SP, Brazil) and α2-adrenergic receptor agonist 2% xylazine hydrochloride (30 mg/kg—Sedanew, Vetnil, São Paulo, SP, Brazil). Subsequently, the lungs were harvested to obtain tissue samples for subsequent assays on oxidative damage (determination of nitrite/nitrate and reduced glutathione—GSH), inflammatory response (myeloperoxidase—MPO—assay and measurement of Th1 (IL-1β) and Th2 (IL-4) cytokine profiles), and histological evaluation (inflammatory score and pulmonary remodeling).

**Figure 2:**
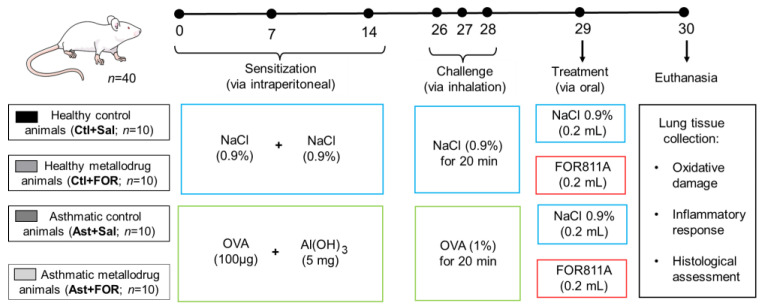
Experimental design in female mice (*Mus musculus*, Swiss strain) to evaluate the effect of a single dose of FOR811A as a rescue drug in acute asthma crises. Four experimental groups were tested, consisting of healthy controls treated with saline (Ctl+Sal) or FOR811A (Ctl+FOR) and asthmatic treated with saline (Ast+Sal) or FOR811A (Ast+FOR). All animals were sensitized on days 0, 7, and 14 with saline solution (0.9% NaCl) or ovalbumin (OVA) dissolved in aluminum hydroxide [Al(OH)_3_]. On days 26, 27, and 28, the animals were challenged with inhalation of 0.9% NaCl or OVA. On day 29, they received a single dose of 0.9% NaCl or FOR811A, and finally, they were euthanized on day 30 for subsequent analysis of lung tissue.

Details of the experimental design are shown in Figure 2.

### 2.4. Assessment of Oxidative Damage

#### 2.4.1. Preparation of Lung Tissue

Lung tissue samples were homogenized (10 times (*w*/*v*) on ice) with 0.1 M phosphate buffer (pH 7.4). The homogenates were centrifuged at 10,000 rpm for 15 min, and aliquots of the supernatants were separated and used to determine oxidative damage parameters.

#### 2.4.2. Determination of Reduced Glutathione (GSH)

GSH levels were quantified to assess endogenous defenses against oxidative stress. To determine GSH (γ-glutamyl-L-cysteinylglycine), the tissue homogenate was prepared with 0.02 M EDTA. Samples were mixed with 50% trichloroacetic acid and centrifuged (3000 RPM, 15 min, 4 °C). The supernatant (400 µL) was transferred and added to 800 µL of Tris-HCl buffer (0.4 M, pH 8.9) and 20 µL of DTNB (5,5′-dithiobis-(2-nitrobenzoic acid)). Finally, absorbance was measured spectrophotometrically at 412 nm (UV ASYS 340, Biochrom, Cambridge, UK). The results are expressed in µg/g of tissue.

#### 2.4.3. Nitrite/Nitrate Assay

Lung tissue was macerated at a ratio of 50 mg in 500 µL of 1.15% potassium chloride (KCl). The homogenate was centrifuged in tubes at a speed of 5000 rpm for 20 min. The supernatant was collected and analyzed for NOx. Nitrite measurement was obtained as an indicator of nitric oxide production through the total determination of nitrite/nitrate (NO_2_^−^/NO_3_^−^) in the lung tissue. The NO_2_^−^ measurement was performed using a colorimetric method based on the Griess reaction. For the experiment, 100 µL of Griess reagent (1% sulfanilamide/0.1% N-(1-naphthyl)ethylenediamine hydrochloride/5% phosphoric acid/distilled water in a 1:1:1:1 ratio) was added to 100 µL of the homogenate supernatant and incubated at room temperature for 10 min. Under acidic conditions, nitrite reacts with sulfanilamide to form an intermediate compound, diazonium salt. This salt then reacts with N-(1-naphthyl)ethylenediamine (NEED) to form a stable purple azo compound, with an absorbance peak at 540 nm.

### 2.5. Evaluation of the Inflammatory Response

#### 2.5.1. Myeloperoxidase (MPO) Assay

Right lung samples (typically 50–100 mg) were carefully excised, weighed, and homogenized in 1 mL of 0.5% hexadecyltrimethylammonium bromide (HTAB) solution in a 50 mM phosphate buffer (pH 6). The samples were homogenized on ice using a tissue homogenizer. The homogenate was then centrifuged at 40,000× *g* for 15 min at 4 °C to remove insoluble material. The resulting supernatant was used for MPO activity assessment. MPO activity was quantified by adding 0.1 mL of the supernatant to 2.9 mL of phosphate buffer (50 mM, pH 6.0) containing 0.167 mg/mL of o-dianisidine hydrochloride and 0.0005% hydrogen peroxide. Absorbance was measured at 460 nm using a spectrophotometer. The MPO activity was determined by measuring the absorbance at 0 and 5 min. The change in absorbance was used to calculate the MPO activity (U) based on the linear equation obtained from an MPO standard curve. The results are expressed as MPO units per milligram of tissue.

#### 2.5.2. Cytokine Measurement of Th1 (IL-1β) and Th2 (IL-4) Profiles

The cytokine levels for IL-1β (Th1) and IL-4 (Th2) were quantified by enzyme-linked immunosorbent assay (ELISA). For the preparation of the lung homogenate, the lung tissue was homogenized in 1 mL of ice-cold phosphate-buffered saline (PBS), followed by centrifugation at 1000× *g* for 10 min at 4 °C. The supernatant was collected and stored at −80 °C until further analysis.

For cytokine measurement, 50 µL of the lung homogenate supernatant was added to each well of a pre-coated ELISA plate (R&D Systems DuoSet kits for IL-1β and IL-4, Minneapolis, MN, USA). The plates were incubated for 2 h at room temperature, followed by washing steps as per the manufacturer’s protocol. The samples were incubated with biotinylated detection antibodies for 1 h, followed by incubation with streptavidin-horseradish peroxidase (HRP) for 30 min. The reaction was developed using tetramethylbenzidine (TMB) substrate, and absorbance was measured at 450 nm using a microplate reader (Diatek, Wuxi, China). The results were calculated using a standard curve and are expressed as cytokine concentrations in picograms per gram of tissue (pg/g).

### 2.6. Histological Analysis

#### 2.6.1. Inflammatory Score Analysis

In the lung tissues, a scoring scale ranging from 0 to 4 was established, considering the degree of tissue compromise, with a score of 0 assigned to tissue with minimal impairment [10]. Inflammatory aspects such as the presence and intensity of cellular infiltrate, vascular dilation and engorgement, edema, and epithelial tissue damage were evaluated and classified according to the standardized scores outlined as follows:

- Score 0: Intact epithelial and connective tissues; no cellular infiltrate; the absence of edema in the mucosa.
- Score 1: Epithelial and connective tissues without vasodilation; no or minimal cellular infiltrate; the absence of edema in the mucosa.
- Score 2: Mild vascular engorgement; areas of mild epithelial desquamation; minimal cellular infiltrate with an increased number of inflammatory cells; the absence of edema in the mucosa.
- Score 3: Moderate vascular engorgement; areas of moderate epithelial desquamation; moderate cellular infiltrate with a predominance of eosinophils; the presence of edema and potential alterations in the mucosa.
- Score 4: Marked vascular engorgement; areas of severe epithelial desquamation; pronounced vasodilation; significant cellular infiltrate with a higher number of eosinophils; the presence of edema and alterations in the mucosa.

#### 2.6.2. Pulmonary Remodeling

Histological sections were stained to quantify collagen and elastic fibers. For collagen, Picrosirius was used [11,12], as follows: incubation in 0.1% Sirius red for 1 h, with ten images per sample (polarized microscope, 40×, Axio Scope.A1, Zeiss, Jena, Germany) in 10 random fields, covering the entire tissue. Collagen predominance (yellow-red type I/yellow-green type III) and fibrosis (scale 1–4: 1 = minimal/absent, 2 = mild, 3 = moderate, and 4 = intense) were evaluated by 2 blinded observers, with average scores. Collagen areas were quantified in Image Pro Plus 5.1 (thresholding, manual selection, area percentage). Elastic fibers were quantified with specific staining, using a similar protocol.

### 2.7. Statistical Analysis

Data analysis was conducted using GraphPad^®^ Prism version 7.0 (GraphPad Software, San Diego, CA, USA). For pulmonary mechanics and scores (inflammatory, remodeling, and peribronchial lesions), a One-Way Analysis of Variance (ANOVA) followed by Bonferroni post-test was employed. For oxidative and inflammatory damage assays, One-Way ANOVA and Tukey post-test were utilized. The results are presented as Mean ± Standard Error of the Mean (SEM). Values of *p* < 0.05 were considered significant.

## 3. Results

### 3.1. Effect of FOR811A on Oxidative Damage

#### 3.1.1. Effect on Reduced Glutathione (GSH) Levels

**Figure 3:**
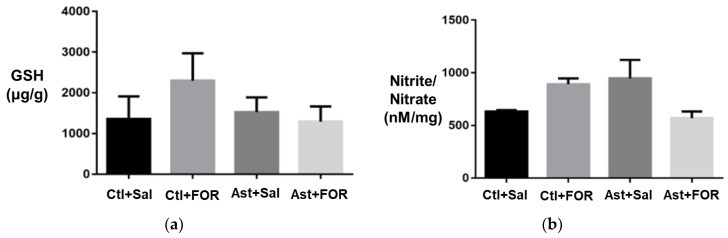
Effect of FOR811A on oxidative damage in lung tissue of the experimental groups [healthy controls treated with saline (Ctl+Sal) or FOR811A (Ctl+FOR) and asthmatic treated with saline (Ast+Sal) or FOR811A (Ast+FOR)]: (**a**) effect on GSH levels (µg/g) and (**b**) effect on nitrite levels (nM/mg). Data are expressed as mean ± standard error of the mean in lung tissue for *n* = 6. Statistical analysis was performed using One-Way ANOVA followed by Tukey’s test (*p* < 0.05).

Treatment with FOR811A (Ast+FOR, 2297.06 ± 672.94 µg/g) did not significantly increase GSH levels compared to the other groups, as follows: 1353.72 ± 516.48 µg/g (Ctl+Sal), 1291.51 ± 345.29 µg/g (Ctl+FOR), and 1526.16 ± 334.01 µg/g (Ast+Sal). GSH levels showed a trend of alteration in the asthmatic group (Ast+Sal) compared to the control group (Ctl+Sal), although this difference did not reach statistical significance. No statistically significant differences were observed in the GSH levels in the pulmonary tissue among the studied groups (Figure 3a).

#### 3.1.2. Effect on Nitrite/Nitrate Levels

Treatment with FOR811A (Ast+FOR, 569.80 ± 62.70 nM/g) did not significantly reduce nitrite/nitrate levels compared to the other groups, as follows: 632.59 ± 13.56 nM/g (Ctl+Sal), 893.53 ± 53.57 nM/g (Ctl+FOR), and 946.56 ± 176.72 nM/g (Ast+Sal). The nitrite/nitrate levels also exhibited a trend of change in the asthmatic group (Ast+Sal) compared to the control group (Ctl+Sal), although without statistical significance. No statistically significant differences were observed in the nitrite/nitrate levels among the studied groups (Figure 3b).

### 3.2. Effect of FOR811A on the Inflammatory Response

#### 3.2.1. Myeloperoxidase Assay

**Figure 4:**
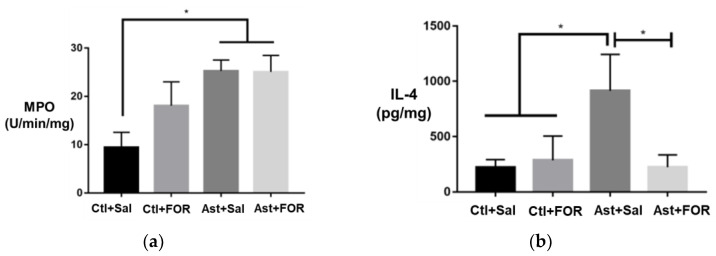

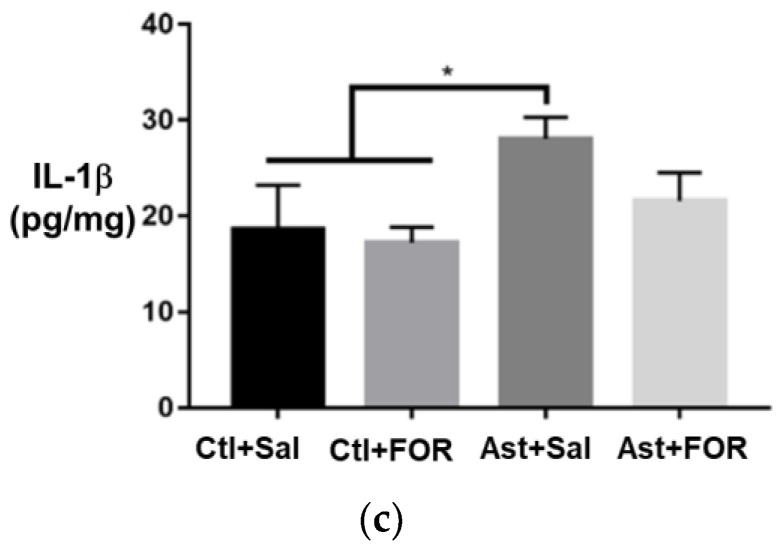
Effect of a single dose of FOR811A on pulmonary inflammatory response in an asthma episode among the experimental groups [healthy controls treated with saline (Ctl+Sal) or FOR811A (Ctl+FOR), and asthmatic groups treated with saline (Ast+Sal) or FOR811A (Ast+FOR)]: (**a**) Myeloperoxidase (MPO) Assay (U/min/mg), (**b**) IL-4 (pg/mg), and (**c**) IL-1β (pg/mg). Data are expressed as mean ± standard error of the mean. Statistical analysis was performed using One-Way ANOVA followed by Tukey’s test (*p* < 0.05, *).

The MPO activity was significantly increased in the asthmatic group treated with saline (Ast+Sal, 25.27 ± 2.24 U/min/mg) compared to the control group (Ctl+Sal, 9.41 ± 3.12 U/min/mg), demonstrating the inflammatory effect of asthma induction. Treatment with FOR811A in the asthmatic group (Ast+FOR, 25.05 ± 3.39 U/min/mg) did not significantly reduce MPO activity compared to the untreated asthmatic group (Ast+Sal). The MPO activity in the FOR811A-treated control group (Ctl+FOR, 19.41 ± 4.48 U/min/mg) was also higher than that the untreated control group (Ctl+Sal), although without statistical significance. These results indicate that FOR811A did not significantly modulate the MPO activity in either the control or asthmatic animals (Figure 4a).

#### 3.2.2. Th2 Profile (IL-4)

The IL-4 levels were significantly increased in the asthmatic group treated with saline (Ast+Sal, 914.05 ± 328.83) compared to the asthmatic group treated with FOR811A (Ast+FOR, 121.16 ± 50.15), indicating that FOR811A treatment significantly reduced the IL-4 levels in asthmatic animals. However, when comparing the asthmatic group treated with saline (Ast+Sal) to the control group treated with saline (Ctl+Sal) or the control group treated with FOR811A (Ctl+FOR), there was no statistically significant difference observed. This suggests that the control groups were similar to each other, and that the main effect was the reduction in IL-4 in the asthmatic animals by FOR811A (Figure 4b).

#### 3.2.3. Th1 Profile (IL-1β)

The IL-1β levels were significantly elevated in the asthmatic group treated with saline (Ast+Sal, 28.06 ± 2.23) compared to both control groups (Ctl+Sal, 18.60 ± 4.62; Ctl+FOR, 17.21 ± 1.64), demonstrating the inflammatory effect of asthma induction. However, treatment with FOR811A in the asthmatic group (Ast+FOR, 21.57 ± 2.93) did not result in a statistically significant reduction in IL-1β levels compared to the untreated asthmatic group (Ast+Sal). This indicates that, while FOR811A did not significantly reduce the IL-1β levels in the asthmatic animals, the asthmatic group showed a significant increase when compared to the control groups (Figure 4c).

### 3.3. Effect of FOR811A on the Inflammatory Score

**Figure 5:**
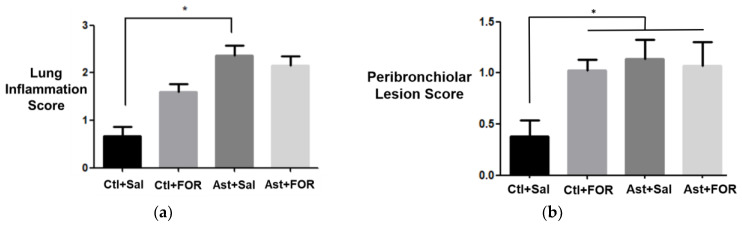
Effect of FOR811A on the inflammatory score in the experimental groups [healthy controls treated with saline (Ctl+Sal) or FOR811A (Ctl+FOR), and asthmatic groups treated with saline (Ast+Sal) or FOR811A (Ast+FOR)]: (**a**) pulmonary inflammation score and (**b**) peribronchiolar pulmonary lesion score. Data are expressed as mean ± standard error of the mean in lung tissue for *n* = 10 (Ctl+Sal; Ctl+FOR; Ast+Sal) and *n* = 9 (Ast+FOR). Statistical analysis was performed using One-Way ANOVA followed by Bonferroni test (*p* < 0.05, *).

A semiquantitative assessment of the cellular inflammatory infiltrate in the lung tissue (Figure 5a) revealed a significant increase in inflammation in the asthmatic group treated with saline (Ast+Sal, 2.367 ± 0.35) compared to the control group treated with saline (Ctl+Sal, 0.675 ± 0.36). However, no statistically significant difference was observed between the groups treated with the metallopharmaceutical (Ctl+FOR, 1.6 ± 0.33; Ast+FOR, 2.15 ± 0.37). In contrast, the analysis of peribronchiolar lesion severity (Figure 5b) revealed a significant difference between the control group treated with saline (Ctl+Sal, 0.38 ± 0.32) and all other groups (Ctl+FOR, 1.02 ± 0.17; Ast+Sal, 1.14 ± 0.37; Ast+FOR, 1.04 ± 0.26). Specifically, the Ctl+Sal group exhibited a significantly lower peribronchiolar lesion severity compared to the other three groups, indicating that FOR811A treatment, regardless of asthma induction, increased peribronchiolar lesion severity.

### 3.4. Analysis of Pulmonary Remodeling Score

**Figure 6:**
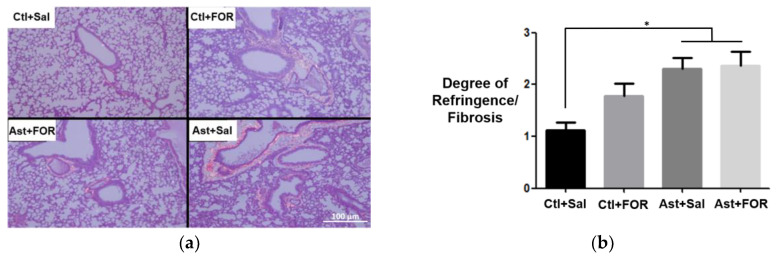

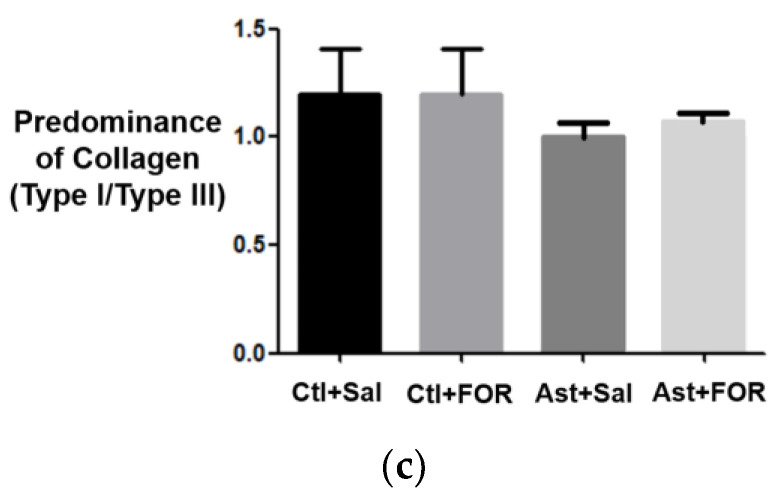
Effect of a single dose of FOR811A on pulmonary remodeling in asthma among the experimental groups [healthy controls treated with saline (Ctl+Sal) or FOR811A (Ctl+FOR) and asthmatics treated with saline (Ast+Sal) or FOR811A (Ast+FOR)]: (**a**) pulmonary histology for semiquantitative evaluation of collagen (40× magnification) and (**b**) score for degree of refringence/fibrosis (1–4), and (**c**) score for collagen predominance (yellow-red type I/yellow-green type III). IL-1β (pg/mg). Data are expressed as mean ± standard error of the mean in lung tissue for *n* = 10 (Ctl+Sal; Ctl+FOR; Ast+Sal) and *n* = 9 (Ast+FOR). Statistical analysis was performed using One-Way ANOVA followed by Bonferroni’s test (*p* < 0.05, *).

Based on a semiquantitative evaluation of collagen in lung histology, the degree of refringence/fibrosis and the predominance of collagen (yellow-red type I/yellow-green type III) in the experimental groups were determined. Picrosirius staining revealed that the healthy control group without treatment (Ctl+Sal) exhibited low collagen deposition, with a predominance of type III collagen. The healthy group treated with the metallopharmaceutical (Ctl+FOR) maintained this pattern, demonstrating that this compound did not alter collagen deposition. In the asthmatic group without treatment (Ast+Sal), a significant increase in collagen deposition was observed, especially type I collagen, indicating tissue remodeling and possible fibrosis. However, the asthmatic group treated with the metallopharmaceutical (Ast+FOR) showed a reduction in collagen deposition compared to Ast+Sal, suggesting an attenuating effect of the treatment on pulmonary fibrosis (Figure 6a). The analysis of the degree of refringence/fibrosis (Figure 6b) revealed a significant difference between the control group treated with saline (Ctl+Sal, 1.125 ± 0.25) and both asthmatic groups (Ast+Sal, 2.3 ± 0.35; Ast+FOR, 2.367 ± 0.45). However, no statistically significant difference was observed between Ctl+Sal and Ctl+FOR (1.783 ± 0.61).

When analyzing the score for collagen predominance (yellow-red type I/yellow-green type III), it was observed that yellow-red type I collagen was predominant and similarly distributed among the experimental groups, with no statistical difference (Figure 6c). Specifically, the scores were as follows: Ctl+Sal (1.2 ± 0.36), Ctl+FOR (1.2 ± 0.36), Ast+Sal (0.96 ± 0.06), and Ast+FOR (0.9 ± 0.1).

## 4. Discussion

As a chronic disease, one of the treatment goals for allergic asthma is to prevent long-term airflow limitation [2]. Nitric oxide (NO) plays a crucial role in asthma treatment by regulating processes such as bronchial relaxation and protecting against bronchoconstriction, which is the excessive contraction of the airways [13]. This occurs because exhaled NO is primarily produced in the nose and paranasal sinuses [13], where it activates soluble guanylate cyclase (sGC), increasing the synthesis of cyclic GMP (cGMP) [8]. cGMP, in turn, activates kinases that perform regulatory functions, such as muscle relaxation, neuronal transmission, the inhibition of platelet aggregation, and the regulation of vascular and bronchial tone, providing protection against excessive bronchoconstriction [13,14]. However, the bronchodilator action of NO in the lungs [15] is limited due to its short half-life and the potential for production deficiencies to be pathological [14].

Substances that activate the NO pathway, such as ruthenium complexes that release NO, are being investigated as potential treatments for asthma, emerging as promising therapeutic agents in the modulation of the NO/sGC/cGMP pathway. In murine models, NO-donating metallodrugs, such as FOR811A, are effective against acute inflammation [16], as well as in allergic asthma models, demonstrating an improved inspiratory capacity, reduced alveolar collapse, and maintained bronchoconstriction [8].

For the first time, we evaluated FOR811A as a single-dose rescue medication for asthma due to its ability to donate NO and assist in regulating the inflammatory and bronchial response. For this, we utilized an allergic airway inflammation protocol in Swiss mice, induced by intraperitoneal sensitization and ovalbumin challenge [1,17]. The ovalbumin (OVA)-induced asthma model was chosen due to its ability to mimic important characteristics of human allergic asthma, such as Th2-cell-mediated airway inflammation, IgE production, and bronchial hyperreactivity [18]. This model is widely used and well-characterized, facilitating comparisons with other studies and standardizing experimental protocols. The murine model was chosen because mice are commonly used in bronchial asthma experiments, as their allergic response mirrors key features of human asthma, including both acute and late phases [17]. Furthermore, this model was selected due to its lack of consanguinity, similar to humans [19], and because female mice are more sensitive to developing allergic inflammation compared to males [20].

Asthma is a heterogeneous inflammatory airway disease with distinct immune endotypes, including Th2-driven responses with eosinophilia and non-Th2 responses with neutrophilia. Inflammation is accompanied by airway remodeling and hyperresponsiveness [21]. Excessive reactive oxygen species (ROS) production by eosinophils and neutrophils, combined with impaired antioxidant defenses, promotes oxidative stress and worsens asthma pathophysiology [21]. Both endogenous and exogenous reactive species, such as superoxide, hydroxyl radicals, hydrogen peroxide, nitric oxide, peroxynitrite, and nitrite, significantly contribute to airway inflammation and determine asthma severity [22]. Although oxidative stress is crucial in asthma, our study shows that FOR811A does not worsen it. As a NO precursor rather than a free radical, FOR811A helps to minimize oxidative damage. The unchanged nitrite/nitrate and GSH levels suggest no exacerbation of oxidative stress, supporting its role in regulating inflammation without further harm.

To defend against oxidative damage caused by these oxidative radicals, the body has an antioxidant protection system [23], with glutathione (GSH) being an example of an antioxidant that acts as a scavenging molecule to protect cells from oxidation and maintain redox homeostasis [24]. The reduced form of GSH is the active form that neutralizes these free radicals [24] by donating electrons to neutralize these harmful molecules, thereby protecting lipids, proteins, and DNA from oxidative damage [23]. Nitrite is often used as a marker for nitric oxide (NO) because, in aqueous solution, NO reacts with molecular oxygen, accumulating in plasma/serum as nitrite (NO_2_^−^) and nitrate (NO_3_^−^) ions [25]. Thus, it serves as an indicator of NO formation and peroxynitrite (ONOO^−^) in tissues.

At the level of oxidative damage, our study showed that the use of FOR811A did not increase reduced GSH levels, suggesting that this metallodrug does not modulate antioxidant responses via GSH. Additionally, no reduction in nitrite/nitrate levels was observed. The levels of nitrite/nitrate were higher than the protective capacity of GSH, indicating that FOR811A was not able to fully protect the pulmonary tissue from the oxidative damage present in asthma. FOR811A is a ruthenium compound that contains a nitrosyl group (NO⁺) in its structure [9], which is chemically distinct from the NO radical, although it acts as a precursor to NO. Considering this chemical structure, it is possible that FOR811A did not reduce the levels of nitrite/nitrate due to an increase in the availability of NO in the pulmonary tissue. These high levels of nitrite/nitrate indicate intense nitrosative stress, which likely overwhelmed any potential GSH-replenishing effects of FOR811A [26].

At the level of the inflammatory response, our findings showed a significant increase in MPO activity in the asthmatic group treated with saline (Ast+Sal) and metallodrug (Ast+FOR) compared to the control group (Ctl+Sal), indicating an inflammatory process associated with this pathological condition. These findings were expected, as the enzyme MPO is released from the primary azurophilic granules of neutrophils, with its levels being higher in individuals with bronchial asthma compared to healthy individuals [27]. However, FOR811A was not able to reduce MPO activity in the asthmatic groups, suggesting that it is ineffective in reducing neutrophil-mediated inflammation in asthma. According to Abu-Soud and Hazen [28], the higher the local concentration of nitric oxide (NO), the lower the MPO activity, as NO modulates MPO’s catalytic activity, which could explain the lack of effect observed in this study.

The main molecular pathways of asthma include T2 inflammation, mediated by Th2 and ILC2 cells, eosinophils, and the cytokines IL-4, IL-5, and IL-13, as well as non-T2 inflammation, mediated by neutrophils, macrophages, and the cytokines IL-1, IL-6, and IL-17. Therapies targeting these pathways and mediators have proven effective in reducing exacerbation and improving lung function in subgroups of patients with severe asthma [29]. In this context, FOR0811A was tested for its potential to modulate inflammation in the cytokines IL-4 (Th2 profile) and IL-1B (Th1 profile).

In the Th2 profile, the asthmatic group treated with saline (Ast+Sal) showed an increase in IL-4 levels compared to the asthmatic group treated with the metallodrug (Ast+FOR). These findings suggest that FOR811A reduced IL-4 release, indicating an anti-inflammatory effect through cytokine modulation and a corresponding decrease in bronchial reactivity to allergic triggers. Our study demonstrates that FOR811A, a nitric oxide donor, was able to reduce the IL-4 levels in an allergic asthma model. This effect may be related to the ability of nitric oxide donors to inhibit IL-4 production, as observed in previous studies [30], suggesting that FOR811A modulates the Th2 inflammatory response in asthma.

In the Th1 profile, the asthmatic group (Ast+Sal) showed higher IL-1β levels compared to the control groups treated with saline (Ctl+Sal) and FOR811A (Ctl+FOR), but no statistical difference was noted between the asthmatic groups. These high levels of IL-1β suggest neutrophil activity, as this cytokine is an important neutrophil activator [31], and, thus, corroborate the data obtained showing an increase in MPO activity. These findings indicate that FOR811A was not able to reduce the oxidative damage and inflammatory injury caused by asthma.

Regarding the lung inflammatory score, the asthmatic groups presented a similar degree of inflammation, with an increased amount (intense degree with scores ranging from two to three), while the control groups showed aspects of normality (mild degree). Similarly, the asthmatic groups exhibited comparable peribronchiolar injury scores. Although there was no statistical difference among the control groups, the Ctl+Sal group had a lower peribronchiolar injury score. Although there were no statistical differences between the groups, the histological analysis for determining the lung remodeling score revealed that both asthmatic groups exhibited a similar degree of birefringence/fibrosis, as well as a predominance of type I and type III collagen. Due to the similarity between the asthmatic groups treated with saline (Ast+Sal) and with FOR811A (Ast+FOR), these findings indicate that the metallodrug was not able to prevent or attenuate lung remodeling and inflammatory injury.

Individuals with asthma exhibit activated Th2 lymphocytes that increase the production of cytokines responsible for initiating and maintaining the inflammatory process, with IL-4, along with IL-5, IL-13, and CCL11 (eotaxin-1), playing important roles in recruiting eosinophils to the tissue [32], resulting in the production of immunoglobulin E (IgE) antibodies. Additionally, IL-4 is also a factor in the proliferation of B lymphocytes and their differentiation into IgE-producing plasma cells [33]. Although FOR811A did not demonstrate satisfactory results in parameters related to oxidative damage, the Th1 inflammatory response, and lung remodeling, the use of a single dose of this metallodrug showed a promising role in modulating Th2 inflammation, due to the significant reduction in IL-4 in the asthmatic group. These findings corroborate the pulmonary mechanics experiments, in which FOR811A reduced tissue and airway resistance [8]. Although the increase in MPO activity observed in the healthy control group treated with FOR811A was not statistically significant, this trend suggests a mild, potentially inflammatory response, which could indicate early leukocyte migration and warrants further investigation. When combined with the present findings of reduced IL-4 levels, FOR811A directly contributed to the decrease in bronchial reactivity related to allergic triggers.

The anti-asthmatic action of FOR811A may stem from its influence on guanylate cyclase (GC) enzymes, specifically at the cysteine residue Cys141 [8], which increases the levels of cyclic guanosine monophosphate (cGMP), thereby reducing the Th2-mediated allergic response. The cGMP produced by the action of GC enzymes [8,34] acts as a second messenger, participating in the signaling and regulation of various cellular functional aspects, as well as modulating smooth muscle relaxation, vascular tone, and decreasing bronchoconstriction [13,14,34]. The activation of GCs and the subsequent increase in cGMP reduce bronchial hyperreactivity in the airways and attenuate the allergic inflammatory response [35], which aligns with the findings regarding FOR811A [8], particularly in single-dose administration through Th2 modulation (IL-4). Consequently, this leads to a decrease in allergic mediators, reduced degranulation, and the formation of reactive oxygen species (ROS), resulting in the relaxation of the airway smooth muscles. Despite the limitations of the OVA-induced asthma model in mice, our findings provide relevant information on acute inflammation and the bronchodilator response, which can be complemented by future studies in more complex models or in patients.

## 5. Conclusions

The use of FOR811A in a single dose reduced the release of IL-4, indicating a decrease in bronchial reactivity and providing a potential benefit in modulating the Th2 inflammatory profile, in addition to promoting smooth muscle relaxation. However, the metallodrug showed inadequate modulation of the antioxidant response, and no reduction in neutrophil-mediated inflammation was observed, as indicated by the elevated levels of IL-1β and MPO, along with a lack of structural improvements in the lungs. These findings suggest that the therapeutic potential of FOR811A as a rescue drug may be limited when administered as a single dose.

## Data Availability

The original contributions presented in the study are included in the article. The data presented in this study are available on request from the corresponding author.
